# The Role of Carvedilol in the Treatment of Dilated and Anthracyclines-Induced Cardiomyopathy

**DOI:** 10.3390/ph4050770

**Published:** 2011-05-24

**Authors:** Kenichi Watanabe, Wawaimuli Arozal, Flori R. Sari, Somasundaram Arumugam, Rajarajan A. Thandavarayan, Kenji Suzuki, Makoto Kodama

**Affiliations:** 1 Department of Clinical Pharmacology, Faculty of Pharmaceutical Sciences, Niigata University of Pharmacy and Applied Life Sciences, Niigata City 956-8603, Japan; 2 Department of Pharmacology, Faculty of Medicine, University of Indonesia, Jakarta 10430, Indonesia; 3 Department of Pharmacology, Faculty of Medicine and Health Sciences, Syarif Hidayatullah Jakarta, State Islamic University, South Jakarta 15412, Indonesia; 4 Department of Gastroenterology and Hepatology, Niigata University Graduate School of Medical and Dental Sciences, Niigata City 951-8510, Japan; 5 First Department of Internal Medicine, Niigata University Graduate School of Medical and Dental Science, Niigata City 951-8510, Japan

**Keywords:** carvedilol, cardiomyopathy, cardioprotection

## Abstract

Although chronic sympathetic activation provides inotropic and chronotropic support to the failing heart, such activation may also have deleterious effects, including the direct cardiotoxic effects of catecholamines, activation of the renin-angiotensin-aldosterone system and an increase in myocardial oxygen demand. These observations indicate that β-blockade might be beneficial in the treatment of heart failure resulting from dilated cardiomyopathy or ischaemic heart disease. Carvedilol is a non-selective β-blocker acting on β1-, β2-, and α1-adrenoceptors. It possesses potent anti-oxidant and anti-apoptotic properties, along with neuroprotective, vasculoprotective, cardioprotective effects, and it has reduced overall mortality in patients with heart failure in controlled clinical trials. Its role in treating cardiomyopathy requires focus. The fact that anthracyclines are cardiotoxic seriously narrows their therapeutic index in cancer therapy. The cardiotoxic risk increases with the cumulative dose and may lead to congestive heart failure and dilated cardiomyopathy in adults and in children. This review focuses on recent research regarding the beneficial effects of carvedilol in the treatment of dilated cardiomyopathy and to revisit the available evidence on the cardioprotection of carvedilol when associated with anthracycline and to explain the mechanisms underlying the benefits of their co-administration.

## 1. Introduction

Cardiomyopathy is a growing public health problem. As the population ages, the incidence of cardiomyopathy is expected to increase greatly. It is a disease of the heart muscle. In most cases, cardiomyopathy causes the heart muscle to become weak, reduce the efficient functioning of the heart muscle, and diminish the ability of the heart to meet the needs of the body. When the heart can no longer pump enough blood to meet the needs of the body, heart failure (HF) is said to be present [1,2].

There are three major types of cardiomyopathy – dilated cardiomyopathy (DCM) [3], hypertrophic cardiomayopathy [4], and restrictive cardiomyopathy [5]. Hypertrophic cardiomyopathy is a genetic disorder that causes a chaotic growth of heart muscle cells within the ventricles and can cause potentially fatal cardiac arrhythmias. Restrictive cardiomyopathy is a very rare condition in which the heart muscle is infiltrated, and made stiff, by abnormal cells, protein, or scar tissue. The common cause is amyloidosis, a disease in which protein-like substance is deposited within the body's tissues and other causes include sarcoidosis and hemochromatosis. DCM is a cardiac muscle disorder characterized by ventricular chamber dilatation and systolic dysfunction, which often leads to HF and sudden death [6,7,8]. In DCM, normal heart muscle becomes damaged, leading to a generalized weakening of the walls of the cardiac chambers leading dilatation of the chambers (remodeling) and congestive heart failure (CHF) [9].

Anthracyclines are some of the most effective drugs currently available in the treatment of neoplastic diseases. However, anthracyclines have profound consequences on the structure and function of the heart causing with time a cardiomyopathy that leads to intractable congestive heart failure [10]. The cardiotoxicity of anthracyclines is dose-dependent and this limits its clinical implementation at optimal antitumor efficacy.

## 2. Causes of DCM

Although the ultimate cause of DCM is unknown, major factors could include diabetes mellitus, coronary artery disease, inflammation of the heart muscle (myocarditis) and alcohol. DCM had been known to result from genetic causes such as mutations in several genes [11,12] or non-genetic insults, such as viruses, alcohol, toxins, and immunological injury [8]. Viral myocarditis always progresses to DCM [13]. Virus-induced myocarditis can result in a dilated hypofunctional heart in animals and is suspected to cause DCM in humans. The onset of unexplained DCM occurring for the first time during the last month of pregnancy or within 5 months after delivery has been termed peripartum cardiomyopathy [14]. Ethanol is known to have dose-related toxic effects on both skeletal muscle and cardiac muscle. Numerous studies have shown that alcohol can depress cardiac contractility and lead to cardiomyopathy [15]. Fatty acid ethyl esters formed from the enzymatic reaction of ethanol with free fatty acids appear to play a role in this disorder. Nutritional abnormalities can also cause cardiomyopathy as evidenced by the significant ventricular impairment associated with some signs of malnutrition [16]. In addition, there are also genetic forms of DCM [17].

## 3. Treatment of DCM

The therapeutic approach to DCM still remains nonspecific and symptomatic, since no specific etiology is identified. The recent introduction of angiotensin converting enzyme (ACE) inhibitors, angiotensin II receptor blocker (ARB) and β-blockers greatly improved the treatment of DCM [18]. Digitalis is added unless contraindicated by adverse effects. Diuretics are helpful in the treatment of CHF associated with DCM [19]. ACE inhibitors and ARB act to dilate blood vessels in the body. ACE inhibitors have proven to be the most effective in improving both the symptoms and the outcome of patients with CHF [20].

## 4. Beta Blockers

It is well known that the sympathetic nervous system is activated during the development of CHF, and its prolonged activation results in ventricular remodeling and progression of cardiac dysfunction [21]. Over expression of β-1 adrenoceptors leads to initial improvement of heart function and ultimately results in HF [22]. Stimulation of β-1 adrenoceptor increases apoptosis [23] and a high level of over expression of β-2 adrenoceptor results in systolic dysfunction and cardiomyopathy [24]. Thus, chronic adrenoceptor activation, which is a compensatory mechanism at initial stages, may exert harmful effects in chronic stages. In spite of some reservations and a great deal of caution, recent years have witnessed a renewed interest in the use of β adrenoceptor antagonists for the treatment of CHF [25].

## 5. Carvedilol in Dilated Cardiomyopathy

Carvedilol is a third-generation vasodilating β blocker that has recently been shown to reduce morbidity and mortality in patients with CHF. This reduction may occur in part through β and α_1_-adrenoceptor blockade, the latter resulting in vasodilation. An extraordinary high degree of cardioprotection beyond that typically seen with beta adrenoceptor blockade suggested that additional properties of the drug might be responsible for this protection. Carvedilol and several of its metabolites are potent antioxidants and it has been shown to exert potent antioxidant effects in multiple cell model systems [26]. In a large, single center CHF population study, carvedilol treated patients have a higher efficacy of ventilation if compared with bisoprolol-treated patients or with CHF patients not receiving beta blockers [27].

Analysis of animal studies reported from our laboratory showed favorable effects of carvedilol in a rat model of DCM induced by autoimmune myocarditis [28,29]. In these reports, low dose carvedilol could improve the cardiac function and reduced the atrial natriuretic peptide, whose level in the plasma is related to the severity of HF [29]. In similar animal model, carvedilol is comparable to losartan in attenuating inflammation, oxidative response, myocardial fibrosis and apoptosis, as well as in preserving energy transcription factors and left ventricular (LV) function [30].

A number of controlled clinical studies showed that carvedilol [31-33] was beneficial to patients with symptomatic CHF due to idiopathic DCM or ischemic disease. Carvedilol also reported to have more beneficial effects in patients with CHF mostly due to ischemic heart disease with idiopathic DCM [34]. Carvedilol was also later reported to have favorable effects in patients exclusively with idiopathic DCM [35]. In COMET trial, 3,029 patients with CHF were randomized to receive metoprolol tartrate (a second-generation beta blocker) with a target dose of 50 mg twice daily or carvedilol with a target dose of 25 mg twice daily. A significant reduction in mortality was observed in favour of the group treated with carvedilol, with an absolute reduction in mortality of 5.7% over a 5 year follow up period, when used to treat CHF mostly due to ischemic heart disease or idiopathic DCM [36]. Recently, a longitudinal cohort study to determine the effects of beta-blockers on outcome patients with Chagas cardiomyopathy with CHF concluded that the mortality was significantly lower in patients taking in comparison to those not taking beta blockers, and patients taking a mean daily dose of carvedilol > or = to 9.375 mg had a marked decrease in mortality in comparison to those not on carvedilol therapy [37]. In addition, in patients with chronic Chagas cardiomyopathy, optimization of treatment with enalapril and spironolactone and subsequent addition of carvedilol 25 mg twice daily were safe and associated with benefits in cardiac function and clinical status [38]. In pediatric patients with dilated cardiomyopathy, carvedilol at a dose of 0.4 mg/kg/day in addition to standard therapy improves cardiac function and symptoms. Carvedilol is well tolerated in children with dilated cardiomyopathy and there is a significant improvement in the clinical status and left ventricular ejection fraction in patients not responding to conventional therapy. In addition there was a case of a child with idiopathic dilated cardiomyopathy and CHF, referred for cardiac transplantation, in whom carvedilol reversed elevated pulmonary vascular resistance [39-41].

## 6. Carvedilol as a Protective Agent against Anthracycline-Induced Cardiac Toxicity

The molecular pathogenesis of anthracycline cardiotoxicity remains highly controversial, although the oxidative stress-based hypothesis involving intramyocardial production of reactive oxygen species (ROS) has gained the widest acceptance [42]. Anthracyclines may promote the formation of ROS through redox cycling of their aglycones as well their anthracycline-iron complexes.

Concomitant use of dexrazoxane, a cardioprotective ion chelating agent, can reduce the risk of this drug-induced cardiomyopathy [43,44]. Dexrazoxane prevents or reduces cardiac injury, as reflected by elevations in troponin T, which is associated with the use of doxorubicin for childhood acute lymphoblastic leukemia without compromising the antileukemic efficacy of doxorubicin [1].

Oliveira *et al.* [45] demonstrated that carvedilol protects cells against doxorubicin toxicity by direct inhibition of exogenous nicotinamide adenine dinucleotide phosphate oxidase (NADPH), the enzyme that involved in the transfer of free electrons to doxorubicin, and ROS formation, thereby preventing oxidative stress and triggering the mitochondrial permeability transition. Carvedilol co-administered with doxorubicin reduces the extent of cell vacuolization in cardiomyocytes, prevents doxorubicin from inhibiting mitochondrial respiration, prevents doxorubicin induced reduction of Ca^2+^ loading capacity and inhibition of respiratory complexes in cardiac mitochondria, and protects against doxorubicin associated lipid peroxidation of cardiac membranes [45].

Similarly, we have reported that DNR rats showed cardiac toxicity as evidenced by worsening cardiac functions, which were evaluated by hemodynamic and echocardiographic studies, malondialdehyde level and the total level of glutathione peroxidase activity in heart tissue. These changes were reversed by treatment with carvedilol, which resulted in significant improvement in the cardiac function. Furthermore, carvedilol down-regulated matrix metalloproteinase (MMP)-2 expression, attenuated the increased protein expression of NADPH oxidase subunits and reduced myocardial apoptosis as well as improved the histopathological changes in heart induced by DNR [46]. A study by Jonsson *et al.* showed that carvedilol increases the cytotoxicity of doxorubicin in tumor cells by inhibiting multiple drug-resistant proteins. This demonstrates that carvedilol does not reduce the effectiveness of anti neoplastics, which is obviously important if it is to be administered together with doxorubicin [47].

The first clinical trial on the prophylactic use of anthracycline-induced cardiomyopathy was performed by Kalay *et al.* In 6-months follow-up study, fifty patients were treated with anthracyclines, 25 of whom also received carvedilol. Co-administration of 12.5 mg carvedilol daily during chemotherapy maintained LV diastolic and systolic function [48]. Accordingly, Mukai *et al.* reported that five cases of severe CHF due to anthracycline-induced cardiomyopathy effectively treated with carvedilol. Their LV functions as well as cardiac symptoms were persistently improved after treatment with carvedilol, suggesting that carvedilol may be an effective therapeutic strategy for anthracycline-induced cardiomyopathy as demonstrated in other forms of CHF [49].

## 7. Carvedilol, Other Than Its Traditional Properties

Inflammatory stresses are cardinal in the pathogenesis of many cardiovascular diseases including diabetic cardiomyopathy, hypertension, atherosclerosis, myocarditis, HF and drug-induced cardiotoxicity. Many treatments for above mentioned disease target the use of anti-inflammation drug which thought to reduce the severity of the disease. Carvedilol has been reported to exert multiple activities, not only anti-oxidant and anti-apoptosis properties, but also anti-inflammation activity [50]. In the context as an anti-inflammation, accumulating evidences have shown that carvedilol has beneficial effect in reducing inflammatory chemokines and cytokines both in experimental and clinical setting of several diseases.

The cytokines hypothesis for HF holds that HF progresses, at least in part, as a result of the toxic effects exerted by endogenous cytokine cascades on the heart and the peripheral circulation [51,52]. Interleukin (IL)-18 is among the pro-inflammatory cytokines involved in cardiovascular changes [53] and serum IL-18/IL-10 ratio is an independent predictor of adverse cardiovascular events in acute coronary syndrome patients [54,55]. Alfieri *et al.* have reported that long-term treatment with carvedilol induced an improvement in symptoms of HF paralleled by an increase of IL-10 levels and a reduction of IL-18 levels, resulting in a 9-times reduction of IL-18/IL-10 ratio and reaching values similar to healthy subjects [52]. However, in this subpopulation of HF patients, inflammation, endothelial function and LV response to carvedilol do not appear to be strictly related [52]. Additionally, in the endothelial cell apoptosis induced by serum of CHF patients, apoptosis of endothelial cells were found to be correlated with elevated tumor necrosis factor (TNF)-α and soluble TNF receptor serum levels [56]. This mechanism has shown that pro-inflammatory cytokines induce endothelial cells apoptosis *in vitro* and carvedilol dose-dependently inhibited the elevated TNF-α-mediated apoptotic process [56]. In DCM, regardless of its etiology, superoxide production and pro-inflammatory cytokines are known to be increased [56]. Carvedilol therapy can significantly preserve LV function, up-regulated Cx43 expression in cardiomyocytes and down-regulate cellular apoptosis in an animal model of DCM by at least partly through the attenuation of pro-inflammatory cytokines and induction of anti-inflammatory markers including IL-10 and endothelial nitric oxide syntheses [30]. A report which has been further investigated the beneficial effect of carvedilol in both ischemic and non-ischemic DCM clarified that carvedilol suppresses the plasma levels of TNF-α and IL-6 in both ischemic and non-ischemic DCM patients. It also improves the functional capacity and the LV ejection fraction significantly in non-ischemic DCM patients and insignificantly in ischemic DCM patients. Overall, carvedilol seems to be more useful in non-ischemic DCM patients than in ischemic patients [57].

Carvedilol has been reported also to reduce the inflammatory-mediated atherogenic process [58]. In the early phase of atherogenesis, cell surface adhesion molecules such as vascular cell adhesion molecule (VCAM)-1 and intercellular cell adhesion molecules (ICAM)-1 could express on endothelial cells to recruit circulating mononuclear cells (MNCs), mainly monocytes, and facilitate their binding to endothelium and migrating to sub-endothelial space [59]. In this report, carvedilol reduced TNF-α-stimulated endothelial adhesiveness to human MNCs by inhibiting intracellular ROS production and VCAM-1, suggesting its potential role in clinical atherosclerosis disease [58].

A broader spectrum of carvedilol beneficial effect has also been confirmed in coxsackievirus-induced and autoimmune-induced myocarditis. The balance between the protective and deleterious immune mechanisms may eventually determine the course of viral myocarditis [60]. Cytokines play a critical role in maintaining this balance as they are known to modulate lymphocyte and myocardial cell functions [61,62]. In the coxsackievirus-induced myocarditis, carvedilol significantly restored the increased level of IL-1β, IL-15 and the decreased level of IL-10 in the infected mice. Moreover, a relevant down-regulation of CD4^+^ T-lymphocytes and an up-regulation of CD8^+^ T-lymphocytes were produced by carvedilol treatment. The significant correlation between IL-1β and MMP-8 mRNA abundance and CD4^+^ T-lymphocytes, as well as the correlation between IL-1β mRNA abundance and hemodynamic parameters emphasize the importance of this cytokine with respect to the MMP/tissue inhibitor of metalloproteinases ratio and hemodynamic function [63]. Therefore, the overall beneficial effect of carvedilol on the LV function observed in the acute phase of coxsackieviral murine myocarditis may be a result of a concerted action of carvedilol on hemodynamic, myocardial inflammation and alteration of the extracellular matrix [63]. In experimental autoimmune myocarditis (EAM), the same beneficial effect of carvedilol in reducing the pro-inflammatory and cytokines has been recently reported. Carvedilol offered protection against EAM; this beneficial cardioprotective effect was attributed to the suppression of the pro-inflammatory cytokines IL-1β and TNF-α, and the promotion of the anti-inflammatory cytokines IL-10 and IL-1RA [64]. This study supported the previous study that superior beneficial effects of carvedilol compared with metoprolol and propranolol in EAM may be partly due to the suppression of inflammatory cytokines [65]. Carvedilol regulated cytokine expression probably *via* the β2-receptor. β2-receptors are expressed on the Th1 T lymphocytes and antigen-presenting cells [66,67].

Thus, it can be concluded that anti-inflammation effect of carvedilol contributes to the clinical efficacy of carvedilol in a broad spectrum of disease and may also confer a range of cardioprotective benefits. However, studies that elucidate by which carvedilol directly regulates the pro-inflammatory chemokines and inflammatory cytokines will be fully required.

## 8. Carvedilol—Precautions and Contraindications

Carvedilol is contraindicated in patients with bronchial asthma or related bronchospastic conditions, decompensated NYHA functional class IV heart failure requiring intravenous inotropic therapy, severe liver impairment, second- or third-degree atrioventricular block, sick sinus syndrome (unless a permanent pacemaker is in place), cardiogenic shock, severe bradycardia or known hypersensitivity to the drug [67,68].

## 9. Conclusions

**Figure 1:**
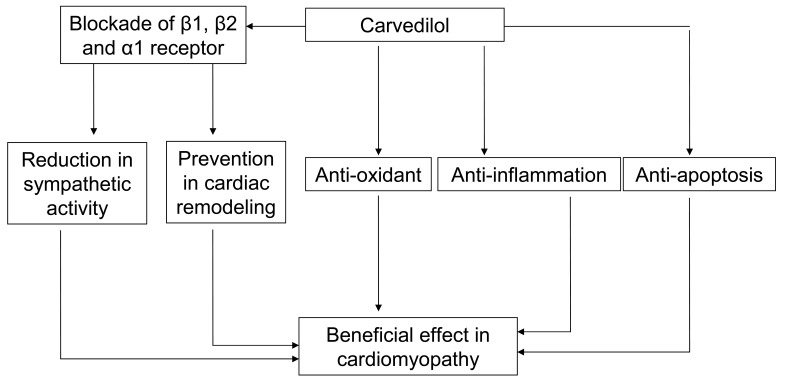
Pathways through which carvedilol exert beneficial effects in cardiomyopathy. Apart from reducing the sympathetic activity, it also has anti-oxidant, anti-apoptosis, and anti-inflammatory action. It can also prevent cardiac remodeling and all of them are responsible for beneficial effects in cardiomyopathy.

The number people with cardiomyopathy increases greatly with respect to time and the therapy for it still remain to be unresolved. Several classes of drugs such as β-blockers, ARB, ACE inhibitors, and diuretics are used in the management of cardiomyopathy. Carvedilol appears to have multiple modes of action, sympathetic blocking, anti-inflammatory, anti-apoptosis, and anti-oxidant activity. It also prevents the cardiac remodeling process proving its role in the treatment of cardiomyopathy (Figure 1).
